# The brain-penetrating CXCR4 antagonist, PRX177561, increases the antitumor effects of bevacizumab and sunitinib in preclinical models of human glioblastoma

**DOI:** 10.1186/s13045-016-0377-8

**Published:** 2017-01-05

**Authors:** Giovanni Luca Gravina, Andrea Mancini, Francesco Marampon, Alessandro Colapietro, Simona Delle Monache, Roberta Sferra, Flora Vitale, Peter J. Richardson, Lee Patient, Stephen Burbidge, Claudio Festuccia

**Affiliations:** 1 Department of Biotechnological and Applied Clinical Sciences, Neurobiology Laboratory, University of L’Aquila, Via vetoios snc, Coppito II, L’Aquila, Italy; 2 Proximagen Ltd., Babraham Research Campus, Cambridge, CB22 3AT UK

**Keywords:** Glioblastoma, CXCR4, Bevacizumab, Sunitinib

## Abstract

**Background:**

Glioblastoma recurrence after treatment with the anti-vascular endothelial growth factor (VEGF) antibody bevacizumab is characterized by a highly infiltrative and malignant behavior that renders surgical excision and chemotherapy ineffective. It has been demonstrated that anti-VEGF/VEGFR therapies control the invasive phenotype and that relapse occurs through the increased activity of CXCR4. We therefore hypothesized that combining bevacizumab or sunitinib with the novel CXCR4 antagonist, PRX177561, would have superior antitumor activity.

**Methods:**

The effects of bevacizumab, sunitinib, and PRX177561 were tested alone or in combination in subcutaneous xenografts of U87MG, U251, and T98G cells as well as on intracranial xenografts of luciferase tagged U87MG cells injected in CD1-nu/nu mice. Animals were randomized to receive vehicle, bevacizumab (4 mg/kg iv every 4 days), sunitinib (40 mg/kg po qd), or PRX177561 (50 mg/kg po qd).

**Results:**

The in vivo experiments demonstrated that bevacizumab and sunitinib increase the in vivo expression of CXCR4, SDF-1α, and TGFβ1. In addition, we demonstrate that the co-administration of the novel brain-penetrating CXCR4 antagonist, PRX177561, with bevacizumab or sunitinib inhibited tumor growth and reduced the inflammation. The combination of PRX177561 with bevacizumab resulted in a synergistic reduction of tumor growth with an increase of disease-free survival (DSF) and overall survival (OS), whereas the combination of PRX177561 with sunitinib showed a mild additive effect.

**Conclusions:**

The CXC4 antagonist PRX177561 may be a valid therapeutic complement to anti-angiogenic therapy, particularly when used in combination with VEGF/VEGFR inhibitors. Therefore, this compound deserves to be considered for future clinical evaluation.

## Background

Glioblastoma (GBM) is the most common malignant brain tumor in adults and makes up approximately 5% of brain tumors in children. Standard of care includes maximal surgical resection of the tumor, followed by radiation in combination with chemotherapy. Despite therapeutic advances over the past decade, the diagnosis of glioblastoma is associated with a median overall survival time of 15–18 months and a 5-year survival rate of less than 5% (see review ref [1]). The failure of standard regimen for GBM can be accounted for by multiple factors including, but not limited to, the heterogeneity of the microenvironment, de novo and/or acquired tumor resistance, and limitations in drug delivery [2]. Alternative approaches, particularly those that can target the mechanisms of recurrence, are required. Therapy failure coupled with the highly vascularized nature of GBM has led to the consideration of agents targeting neo-angiogenesis as alternative therapeutic strategies for this disease (see review [3]). Tumor angiogenesis is strongly regulated by the VEGF/VEGFR system. Bevacizumab, an anti-vascular endothelial growth factor (VEGF) antibody, was approved in the USA in 2009 to treat glioblastoma recurrence on the basis of encouraging preclinical and clinical results [4–6]. Although there is evidence that bevacizumab reduces tumor edema, angiogenesis, and disease burden, the use of this agent as well as other VEGF/VEGFR-targeting drugs has been followed by resistance (largely as a result of adaptive tumor responses) in preclinical models and in clinical settings. Multiple not mutually exclusive hypotheses attempting to explain this resistance include direct effects on glioblastoma cells, modifications of the perivascular niche [12], increased bevacizumab-mediated hypoxia [13, 14], which increases the triggering of proliferation activation in cancer stem cells, and the recruitment of circulating monocytes and macrophages [15] which maintains vessel integrity and cancer stem cell growth. In addition, magnetic resonance imaging (MRI) performed on patients with bevacizumab-resistant glioblastoma revealed that these tumors have more diffuse borders compared to responding tumors. This makes tumor borders difficult to identify and suggests that resistance to bevacizumab may be characterized by a more invasive state [10, 11]. Despite this, data from clinical trials suggest that, in some patients with recurrent GBM treated with bevacizumab or tyrosine kinase inhibitors (e.g., sunitinib [7] which targets VEGFR, c-kit, and PDGFR), improved 6-month progression-free survival rate (PFS) and radio-graphic responses [8–11] can be observed.

Macrophages constitute one of the largest cell populations in glioblastoma [15, 16], and recruitment of myeloid cells [17] has been associated with poor responses to therapy, disease recurrence, and the development of acquired resistance to therapies, including anti-angiogenic strategies [15, 16]. It has been demonstrated that Tie2-expressing monocytes (TEMs), a subpopulation of circulating blood monocytes, previously identified as pro-angiogenic and immunosuppressive, are overrepresented at the invasive front in surgical samples of human glioblastoma and murine glioma models after anti-VEGF therapy [17]. This condition leads to an inflammatory and pro-invasive tumor microenvironment. Recently, it has been reported that inhibition of angiopoietin 2 overrides the heightened monocyte invasion induced by anti-angiogenic therapies in gliomas [16, 17]. In addition, clinical studies indicate that the levels of circulating cytokines are increased in most cancer patients who have undergone anti-VEGF therapy.

Recent experimental data suggest that HGF/MET [18], TGFβ/TGFβR [19], and CXCL12/CXCR4 [20, 21] enhance the invasive phenotype of GBM after anti-VEGF/VEGFR therapy and VEGFR inhibitors may up-regulate CXCR4 in a TGFβR signaling-dependent manner [22]. CXCR4 is a well-known G-protein-coupled receptor (GPCR) for the small chemokine stromal-derived factor (SDF)-1α, which is also known as CXCL12. A second receptor for SDF1α is CXCR7 which is expressed on vascular endothelial cells, T cells, dendritic cells, B cells, brain-derived cells, and tumor cells, including human glioma cells [23, 24]. CXCR4 is implicated in neo-angiogenesis and vasculogenesis, so we hypothesized that combining anti-angiogenic therapy with CXCR4 inhibition would have superior antitumor activity compared to single treatments. We employed the tyrosine kinase inhibitor sunitinib and bevacizumab the anti-VEGF antibody which is selective for human VEGF and shows low affinity for mouse VEGF. Hence, the efficacy of bevacizumab in these experiments is due solely to the sequestration of human tumor cell line-derived VEGF and not that derived from mouse stromal cells.

## Methods

### Cell lines

Human glioma cell lines U251, U87MG, and T98G were originally obtained from the American Type Culture Collection (ATCC, Rockville, MD). A172 and luciferase transfected U87MG cells were kindly provided by Jari E. Heikkila (Department of Biochemistry and Pharmacy, Abo Akademi University, Turku, Finland). Cells were cultured at 37 °C in 5% CO_2_ and were maintained in DMEM containing 10% (*v*/*v*) fetal bovine serum, 4 mM glutamine, 100 IU/ml penicillin, 100 μg/ml streptomycin, and 1% nonessential amino acids (Invitrogen Life Technologies, Inc., Rockville, MD). To minimize the risk of working with misidentified and/or contaminated cell lines, the cells used in studies reported here were stocked at very low passages and used at <20 subcultures. Periodically, DNA profiling by GenePrint® 10 System (Promega Corporation, Madison, WI) was carried out to authenticate cell cultures.

### Chemicals and other reagents

All the materials for tissue culture were purchased from Hyclone (Cramlington, NE, USA). Plasticware was obtained from Nunc (Roskilde, Denmark). Antibodies for β-actin [sc-130065], Ang2 (F1) [sc-74403], Ang-1 (C-19) [sc-6320], MMP-2 [4D3, sc-53630], TGFβ RI (V-22) [sc-398], and CXCR4 [4G10, sc-53534] were purchased from Santa Cruz (Santa Cruz, CA, USA). Tie2 (AB33, Mouse mAb #4224) and Phospho-Tie2 (Ser1119, Antibody #4226) were purchased from Cell Signaling Technology Europe, B.V. (Leiden, The Nederland). PRX177561 was provided by Proximagen Ltd. and is a highly selective CXCR4 antagonist (Ki at human and mouse CXCR4 receptor approximately 3 nM), which shows no activity at the other chemokine receptors, nor at 75 other drug targets (enzymes, receptors, and ion channels) at 10 μM. This compound crosses the blood-brain barrier.

### Matrigel assay

The Matrigel (BD Biosciences, 356237) endothelial branching morphogenesis assay establishes the potential of ECs to form tubular networks. For this, 300 μl Matrigel (10.4 mg/ml, not diluted) was added to the wells of 24-well plates and allowed to gel at 37 °C for 30 min. Then 40,000 hBMEVC (human brain microvascular endothelial cells, kindly provided by Philip M. Cummins, School of Biotechnology, Dublin City University, Ireland, were added to each well and allowed to invade the material for 6–24 h. The endothelial cell line was routinely grown in EndoGRO MV Basal Medium (Millipore, Italia) supplemented with 5% fetal bovine serum, l-glutamine (10 mM), ascorbic acid (50 μg/ml), heparin (0.75 U/ml), hydrocortisone (1 μg/ml), recombinant human epidermal growth factor (5 ng/ml), EndoGRO-LS Supplement (0.2%), and antibiotics (100 μg/ml Mycozap).

### Cell viability assay

The cytotoxicity of bevacizumab, sunitinib, and/or PRX177561 was measured by the Cell Counting Kit-8 (CCK-8; Dojindo Molecular Technologies Inc., Tokyo, Japan). CCK-8 contains Dojindo’s highly water-soluble tetrazolium salt (WST-8), which produces a water-soluble formazan dye upon reduction in the presence of an electron mediator. U87MG, U251MG, and T98G cells were seeded in 96-well plates at a density of 4 × 10^3^ cells per well to allow for adhesion overnight. After this, the cells were treated with different concentration of drugs. After 3 days, 10 μl of the CCK-8 solution was added to each well of the plate, and the plate was incubated for 3 h in the incubator (37 °C; 5% CO_2_). The optical density (OD) of the sample plate was measured at 450 nm in a microplate reader.

### ELISA determinations

After appropriate treatments, tumor cell cultures, tissue extracts, and plasma samples were harvested for the analysis of cytokine and receptor expression. CXCR4 (Cyto Glow CXCR4 [pSer339]), cell-based ELISA, was purchased from Assay Biotech (Sunnyvale, USA). Human SDF1α (Quantikine ELISA Kit) were purchased from R&D systems (Minneapolis, USA). All determinations were performed in triplicate, according to manufacturer’s instructions. Data are presented mean ± standard error (SE). Cytokine levels were normalized to total protein concentration in tissue lysates.

### Mouse glioblastoma xenograft model

Female CD1-nu/nu mice, at 6 weeks of age, were purchased from Charles River (Milan, Italy) and maintained under the guidelines established by our Institution (University of L’Aquila, Medical School and Science and Technology School Board Regulations, complying with the Italian government regulation n.116 January 27 1992 for the use of laboratory animals). All mice received subcutaneous flank injections of 1 × 10^6^ U87MG, U251, and T98G cells representing models for MGMT negative (U87MG, U251MG) and MGMT positive (T98G) cells. Tumor growth was assessed bi-weekly by measuring tumor diameters with a Vernier caliper (length × width). Tumor weight was calculated according to the formula: TW (mg) = tumor volume (mm^3^) = *d* ^2^ × *D*/2, where *d* and *D* are the shortest and longest diameters, respectively. The effects of the treatments were examined as previously described [25]. Mice with tumor volumes of 100–150 mm^3^ were randomized to receive vehicle, bevacizumab (4 mg/kg iv every 4 days), sunitinib (40 mg/kg po qd), or PRX177561 (50 mg/kg po qd), or combinations of bevacizumab and sunitinib with PRX177561. Vehicle was a mixture of hydroxyl-propyl-β-cyclodextrin (HPβCD) at 10% in water (pH7) and propylene-glycol (PG), 25/75 (*w*/*w*). Animals were sacrificed by carbon dioxide inhalation, and tumors were subsequently removed surgically. A part of the tumor was directly frozen in liquid nitrogen for protein analysis, and the other part was fixed in paraformaldehyde overnight for immunohistochemical analyses. Indirect immunoperoxidase staining of tumor xenograft samples was performed on paraffin-embedded tissue sections (4 μm). Briefly, sections were incubated with primary antibodies overnight at 4 °C. Next, avidin–biotin assays was done. Mayer’s hematoxylin was used as nuclear counterstaining. Tumor microvessels were counted at ×400 in five arbitrarily selected fields, and the data were presented as number of CD31^+^ microvessels/×100 microscopic field for each group. Ki67 labeling index was determined by counting 500 cells at ×100 and determining the percentage of cells staining positively for Ki67. Apoptosis was measured as the percentage of tunnel positive cells +/− SD measured on five random fields (×400) on immunofluorescence (IF) images. The presence of red cells in tumor tissue and in blood vessels as well as the presence of microthrombi and bleeding zones was demonstrated by Martius yellow-brilliant crystal scarlet blue technique. Tumor hemoglobin levels were also quantified [25].

### Evaluation of treatment response in vivo (subcutaneous xenograft model)

In order to get closer to the parameters used to analyze the pharmacological efficacy assessments in the man, we quantified the antitumor effects of different treatments as previously described [26–29]: (1) tumor volume, measured throughout the experiment; (2) tumor weight, measured at the end of experiment; (3) complete response (CR) defined as the disappearance of the tumor; (4) partial response (PR) defined as a reduction of greater than 50% of tumor volume with respect to baseline; (5) stable disease (SD) defined as a reduction of less than 50% or an increase of less than 100% of tumor volume with respect to baseline; (6) tumor progression (TP) defined as an increase of greater than 50% of tumor volume with respect to baseline; and (7) time to progression (TTP). These modalities of analysis reduced both the differences of single tumor volume measurements in the time linked to differences of engraftment efficacy of the tumor cells as well as the individual variability of the response (even though the mice were inbreed). Combination index of dual administrations was calculated accordingly Bruzzese et al. [30].

### Dissociation of U87MG xenografts into single-cell suspension

Tumors were removed in a sterile condition from mice and washed two to three times with 5–10 ml of PBS/DMEM basal medium to remove blood and debris. Tissues were cut into small pieces and minced with a scalpel blade into tiny pieces to increase the surface area for trypsinization process. Minced tissues were trypsinized in 3–5 ml of pre-warmed %0.05 trypsin-EDTA for 10–15 min at a 37 °C water bath. After digestion, an equal volume of soybean trypsin inhibitor is added to stop the enzymatic trypsin reaction. Trypsin inactivation is ensured by pipetting the suspension up and down several times. Then, the suspension was pelleted down by centrifuging at 800 rpm (110 g) for 5 min, and supernatant discarded whereas tissue pieces were resuspended in 1 ml of sterile DMEM basal medium. The clumps were dissociated by gently pipetting up and down (three to seven times) until a smooth milky single-cell suspension was achieved. The number of pipetting steps directly depends on the size of particles in the minced tissue. Lengthy and vigorous mechanical dissociation should be avoided as it might result in cell death and a reduction in sphere formation. Next, suspension was pelleted and washed to remove un-dissociated pieces and debris: Cell suspension was filtered through a 40-μm cell strainer into a 50-ml tube. And pelleted cells were counted and used for new passage in nude mice and for CXCR4 expression in western blot.

### Orthotopic intra-brain model

Following IACUC guidelines in an approved animal-use protocol, nude mice were inoculated intracerebrally as follows [31]. Animals were anesthetized with 100 mg/kg ketamine and 15 mg/kg xylazine. The surgical zone was swabbed with Betadine solution, and the eyes were coated with Lacri-lube. The head was fixed in a stereotactic frame (mouse stereotaxic instrument, Stoelting Europe, Dublin, Ireland), and a midline scalp incision was made. A small hole was made at 1.0 mm anterior and 2 mm lateral to the exposed bregma. A sterile 5-μl Hamilton syringe with a 26-gauge needle was inserted to a depth of 3.0 mm from the skull surface and withdrawn by 0.5 mm to inject 3 × 10^3^ U87MG cells in a volume of 3 μl. The injection rate was set to 1 μl/min. After the implantation of the tumor cells, the needle was left in place for 5 min to prevent reflux. The needle was then completely withdrawn from the brain over the course of 4 min (1.0 mm/min), and the skin was sutured. Treatments were started 5 days after cell injection when no luciferase activity was intracranially detectable. Generally, the first positive mouse images were obtained from 20 days following intracranial inoculation. Mice were euthanized when they displayed neurological signs (e.g., altered gait, tremors/seizures, lethargy) or weight loss of 20% or greater of presurgical weight. Blood samples were collected for plasma analysis. All mice were perfused with PBS; a subset was fixed with 4% paraformaldehyde. If fixed, the brains were stored in paraformaldehyde for 24 h, 15% sucrose for 24 h, and then 30% sucrose for 24 h. The brains were paraffin embedded.

### Bioluminescence imaging

Bioluminescence imaging (BLI) was performed by using the Alliance Mini HD6 (UVItec Limited, Cambridge, UK). Briefly, after injection with 150 ug/g d-luciferin (Synchem UG & Co. Altenburg, Germany) in PBS (i.p.), animals were anesthetized with 100 mg/kg ketamine and 15 mg/kg xylazine and analyzed for BLI using the Alliance Mini HD6 machine.

### Statistics

Continuous variables were summarized as mean and standard deviation (SD) or as median and 95% CI. For continuous variables not normally distributed, statistical comparisons between control and treated groups were established by carrying out the Kruskal-Wallis tests. When the Kruskal-Wallis test revealed a statistical difference, pair-wise comparisons were made by Dwass-Steel-Chritchlow-Fligner method and the probability of each presumed “non-difference” was indicated. For continuous variables normally distributed, statistical comparisons between control and treated groups were established by carrying out the ANOVA test or by Student *t* test for unpaired data (for two comparisons). When ANOVA test revealed a statistical difference, pair-wise comparisons were made by Tukey’s Honestly Significant Difference (HSD) test and the probability of each presumed “non-difference” was indicated. Dichotomous variables were summarized by absolute and/or relative frequencies. For dichotomous variables, statistical comparisons between control and treated groups were established by carrying out the exact Fisher’s test. For multiple comparisons, the level of significance was corrected by multiplying the *P* value by the number of comparisons performed (*n*) according to Bonferroni correction. TTP was analyzed by Kaplan-Meier curves and Gehan’s generalized Wilcoxon test. When more than two survival curves were compared, the logrank test for trend was used. This tests the probability that there is a trend in survival scores across the groups. All tests were two-sided and were determined by Monte Carlo significance. *P* values <0.05 were considered statistically significant. SPSS® (statistical analysis software package) version 10.0 and StatDirect (version. 2.3.3., StatDirect Ltd.) were used for statistical analysis and graphic presentation. We analyzed Kaplan-Meier curves [26, 32] in terms of hazard ratios (HRs). This parameter is an expression of the hazard or chance of events occurring in the treatment arm as a ratio of the hazard of the events occurring in the control arm. A hazard ratio of 2 indicates that treatment of reference is twice more effective with respect to a control population.

## Results

### Anti-angiogenic therapies induce the expression of CXCR4 and SDF1α in experimental glioblastomas

**Fig. 1:**
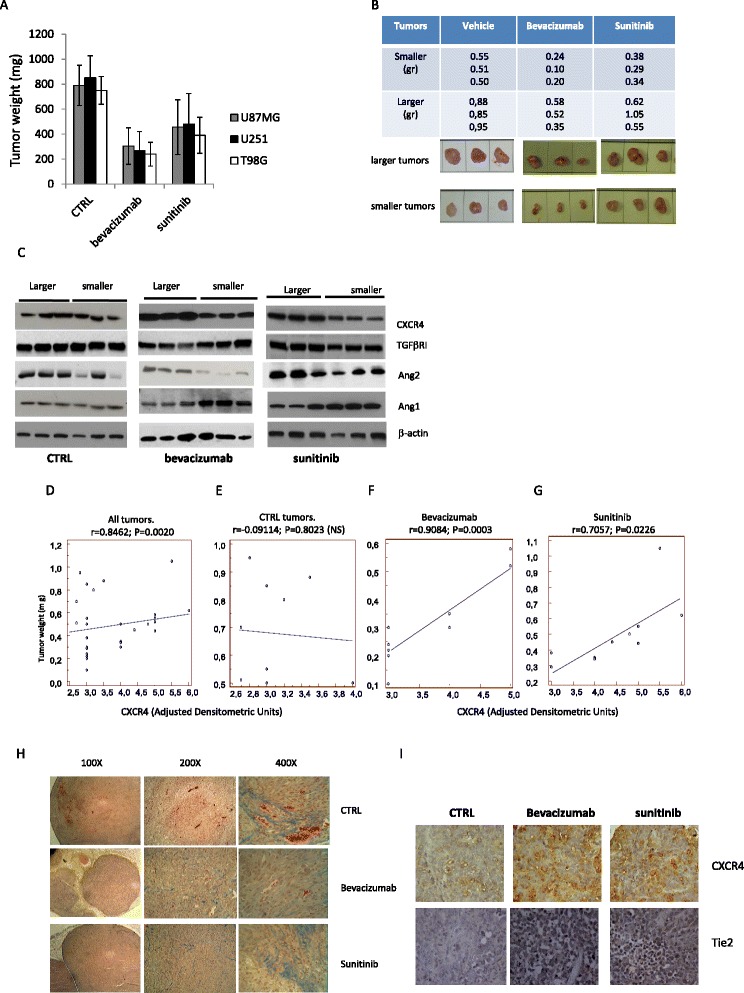
Anti-angiogenic therapies induce the expression of CXCR4 and SDF1α in experimental glioblastomas (1). **a** Graphical distribution of tumor weights at the end of treatment cycle of 35 days (mean ± standard deviation, (SD)). Each column included ten tumors. **b** Size-based grouping of representative U87-derived tumors from animals of control (three tumors) or treated (three tumors) with sunitinib or bevacizumab. **c** Western blot evaluation of single three tissue extracts from smaller and larger tumors in each group of treatment. Each lane was loaded with 100 μg of protein. CXCR4, TGFβRI, Ang2, and Ang1 expression was normalized versus actin. **d** Statistical analyses of correlation performed among the levels of CXCR4 (adjusted densitometric units by western blots normalized versus actin) and tumor weight for all 30 tissues (ten tissue extracts for three treatments). **e** Statistical analyses of correlation performed on untreated tumors (ten tissue extracts). **f** Statistical analyses of correlation performed on bevacizumab treated tumors (ten tissue extracts). **g** Statistical analyses of correlation performed on sunitinib treated tumors (ten tissue extracts). **h**, **i** Histological appearance (**h**) and CXCR4 and Tie2 expression (**i**) in untreated and treated with bevacizumab or sunitinib U87 xenografts with resolution of ×100, ×200, and ×400

**Fig. 2:**
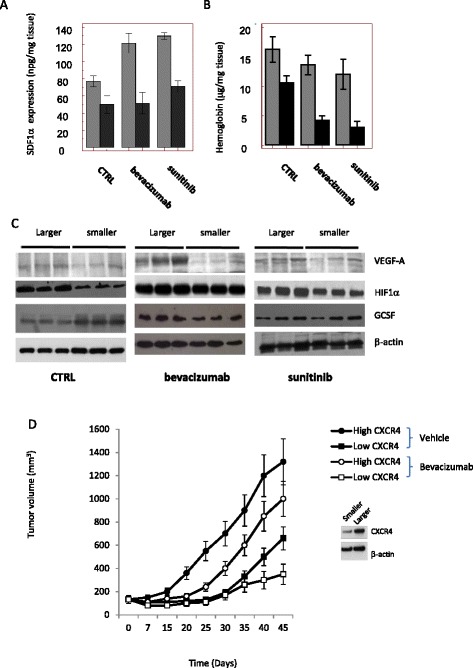
Expression of tumor markers. **a** Expression of SDF1α levels after treatments and differences with tumor size. Analyses of linear regression performed in the sunitinib and bevacizumab treated tumors showed a significant correlation in these groups (*r* = 0.844, *P* = 0.0002). **b** Hemoglobin content in treated and control tumors analyzed according to tumor size. Analyses of linear regression performed in the sunitinib and bevacizumab treated tumors showed *r* = 0.69 and *P* = 0.016. **c** Western blotting determination of VEGF-A, HIF1α, and GCSF. **d** Growth curved derived from U87MG cell suspensions obtained from U87MG xenografts treated for 35 days with bevacizumab and scored as indicated in the text in high CXCR4 expression (cell suspensions from larger tumors) and low CXCR4 expression (cell suspensions from smaller tumors) and grown for 45 days with or without bevacizumab

It has been demonstrated that bevacizumab failure and recurrence show typical malignant behavior in humans with sarcomatous, spindle cell morphology, mitotic figures, and necrosis [33, 34]. Bevacizumab failure is also associated with increased expression and activity of the CXCR4/SDSF1α pathway [35]. To verify if in vivo administration of bevacizumab or sunitinib increased CXCR4/SDSF1α signaling, we treated female nude mice-bearing U87MG, U251, and T98G subcutaneous xenografts with bevacizumab (4 mg/kg iv every 4 days [36]) or sunitinib (40 mg/kg po qd, [37]). After 35 days of treatments, animals were sacrificed and tumor harvested. Half of the tissues were paraffin embedded while the other half used for tissue extract preparations and frozen at −80 °C until use. Immunohistochemical and ELISA determinations were performed in tissue extracts and blood samples. In U87MG cells, we find that bevacizumab and sunitinib reduced tumor weights by about 62 and 42%, respectively (Fig. 1a). Similar percentage changes were found in U251 (69 and 43%, respectively) and T98G (68 and 48%, respectively), although there was a considerable heterogeneity in the size of the tumors after treatment with bevacizumab and sunitinib, suggesting variability in the therapy response in different animals. It is, indeed, possible that larger tumors in the treated groups were less susceptible to anti-angiogenic treatment. So we verified if bevacizumab or sunitinib administration modified the levels of CXCR4, TGFβ, and ang2 and if this was related to the size of the tumors. As shown in the western blotting shown in Fig. 1c, no correlation was found between tumor size and CXCR4 and expression in untreated tumors whereas treatment with bevacizumab or sunitinib seemed to cause an increase in the expression of CXCR4. The statistical analyses of correlation confirmed this qualitative appearance, indicating that no correlation was found in untreated tumors (Fig. 1e) whereas a correlation was observed in treated animals with bevacizumab and sunitinib (Fig. 1f, g) with correlation coefficients of 0.9084 (*P* = 0.0003) and 0.7054 (*P* = 0.0226), respectively. Bevacizumab (*r* = 0.8247, *P* = 0.0054) and sunitinib (*r* = 0.8954; *P* = 0.0033) also caused an increase in TGF-β expression in the larger tumors. We observed also that Ang 2 expression correlated with tumor size in the bevacizumab (*r* = 0.6904; *P* = 0.0287) and sunitinib (*r* = 0.5807; *P* = 0.0388) treated tumors. This suggested that high CXCR4, TGF-β1, and Ang2 expression levels might be associated with reduced sensitivity to these treatments. Increased expression of CXCR4 and Tie2 was confirmed by immunohistochemistry in the larger tumors (Fig. 1i). In Fig. 1h, we show the histological appearance of untreated and treated with bevacizumab or sunitinib U87 xenografts with resolution of ×100, ×200, and ×400). We observed that angiogenesis (confirmed by vessel count or hemoglobin content in Fig. 2b) was higher in CTRL tumors when compared to bevacizumab- or sunitinib-treated tumors. In sunitinib treated tumors, we observed also a significant accumulation of fibrous stroma as response to increased cell death especially in the smaller tumor. In parallel, SDF1α levels were increased by anti-angiogenic therapies but only in the larger tumors (Fig. 2a). Analyses of correlation performed in the sunitinib and bevacizumab treated tumors showed a significant correlation with *r* = 0.844, *P* < 0.001 and *r* = 0.7899, *P* = 0.0007, respectively, suggesting that high SDF1α expression was associated with resistance to angiogenic-based therapy. In order to determine whether the increased expression of CXCR4 and SDF1α was related to the relative resistance to anti-angiogenic treatment, we analyzed the levels of hemoglobin as an indirect marker for increased/decreased angiogenesis. Also in this case, smaller tumors possessed lower hemoglobin content when compared to larger tumors (Fig. 2b) and these differences were higher in treated tumors, with a positive and significant regression coefficients (*r* = 0.6945, *P* = 0.016 and *r* = 0.7932, *P* = 0.0005) respectively in bevacizumab and sunitinib groups indicating once again a strong correlation between angiogenesis and sensitivity to therapy.

In addition, GBM xenografts treated with bevacizumab or sunitinib showed an unusual increase in the production of VEGF and an elevated expression of HIF-1α (Fig. 2c) with little difference between smaller or larger tumors. This is in agreement with that observed with the VEGFR inhibitor PTK787 (vatalanib) [38].

Then, we asked ourselves if the differences in tumor weight could be due to differences in tumor engraftment efficiency. To address this question, we first evaluated if there were differences in the randomization of tumors. As stated in MM, mice were randomized to receive treatments only when tumor volumes reached 100–150 mm^3^. The statistical analyses performed in the different group of tumor, demonstrated that randomization was balanced in terms of initial volumes. No significant difference was, indeed, observed among the groups [controls (130 mm^3^ +/− 14, Mean ± SD), sunitibib (137 mm^3^ +/− 16), and bevacizumab (128 mm^3^ +/− 15)] with respect to tumor volumes at the start of treatments. In order to investigate in more detail the relationship between CXCR4 expression and bevacizumab efficacy, a specific experiment was performed. U87MG xenografted animals were treated with bevacizumab (4 mg/kg iv every 4 days for 35 days). At the end of treatment, tumors were harvested, weighed, and defined as larger (>338 mg) or smaller (<220 mg) tumors accordingly with tumor volumes superior or inferior to 75th and 25th percentiles, respectively. Three out of ten tumors were considered larger and four out of ten tumors were considered smaller. These tumors were then digested to obtain a cell suspension, and the expression of CXCR4 was assessed in smaller and larger tumors generated after bevacizumab treatment. As shown in Fig. 2d, smaller tumors expressed lower CXCR4 levels than larger tumors. For this reason, ten mice for groups received subcutaneous flank injections of 2 × 10^6^ cell suspension deriving from smaller and larger tumors were considered to receive cells with higher or lower CXCR4 expression levels. When tumor volumes reached 100–150 mm3, mice were randomized to receive bevacizumab or vehicle for 45 days. Here, we show that tumor xenograft derived from cellular suspension of larger tumors with higher CXCR4 generated under the first bevacizumab treatment was less responsive to the subsequent treatment with bevacizumab and grown much more of tumor originated from smaller tumors with lower CXCR4. Therefore, this experiment suggests that the amount of CXCR4 expressed in larger tumors may be involved reduced efficacy of tumor cells to bevacizumab treatment.

### In vivo effects of CXCR4 antagonist, PRX177561, alone or in combination with bevacizumab or sunitinib: TTP evaluation

**Fig. 3:**
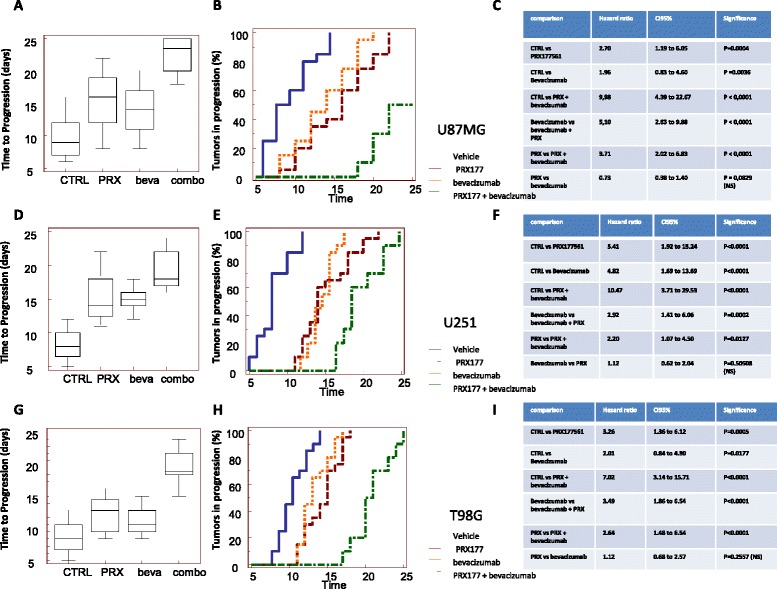
TTP (time to progression) values for control (CTRL), PRX177561, bevacizumab, and the combination in U87MG tumors (**a**), U251 tumors (**d**), and T98G tumors (**g**). Kaplan-Meier estimates for rates of progression in U87MG tumors (**b**), U251 tumors (**e**), and T98G tumors (**h**). Statistical analyses for U87MG tumors (**c**), U251 tumors (**f**), and T98G tumors (**i**). U87MG, U251, and T98G bearing nude mice were treated with bevacizumab (4 mg/kg iv every 4 days) alone or in combination with or PRX177561 (50 mg/kg po qd) in subcutaneous xenograft models. Tumors were randomized when they reached a volume of 100–150 mm^3^ and treated for 35 days. Tumors were measured every 2 days for a total of 17 measurements. After 35 days, animals were sacrificed and tumors harvested and weighed

**Fig. 4:**
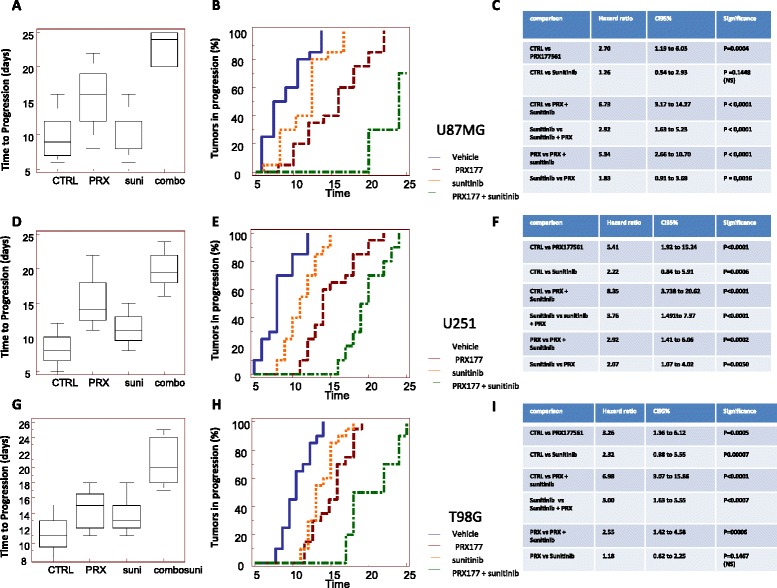
TTP (time to progression) values for control (CTRL), PRX177561, sunitinib (Suni), and the combination in U87MG tumors (**a**), U251 tumors (**d**), and T98G tumors (**g**). Kaplan-Meier estimates for rates of progression in U87MG tumors (**b**), U251 tumors (**e**), and T98G tumors (**h**). Statistical analyses for U87MG tumors (**c**), U251 tumors (**f**), and T98G tumors (**i**). U87MG, U251, and T98G bearing nude mice were treated with sunitinib (40 mg/kg po qd) alone or in combination with or PRX177561 (50 mg/kg po qd) in subcutaneous xenograft models. Tumors were randomized when they reached a volume of 100–150 mm^3^ and treated for 35 days. Tumors were measured every 2 days for a total of 17 measurements. After 35 days, animals were sacrificed and tumors were harvested and weighed

**Fig. 5:**
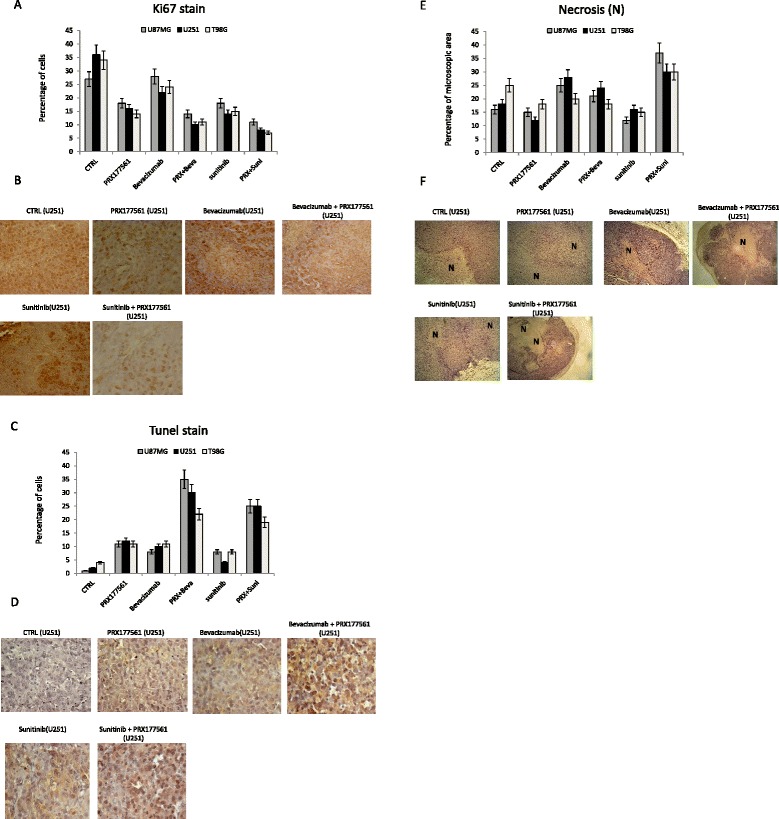
**a** Histological Ki67 staining. Graphical analyses performed in U87MG, U251, and T98G cells. **b** Ki67 staining. Representative images obtained from U251 xenografts. Pictures were not counterstained. **c** TUNEL staining. Graphical analyses performed in U87MG, U251, and T98G cells. **d** TUNEL staining. **e** Palisading necrosis (graphical analyses perfromed in U87MG, U251 and T98G), **f** Representative images in immunohystochemistry obtained from U251 xenografts. Representative images in immunofluorescence obtained from U251 xenografts

Next, we wanted to assess whether the CXCR4 antagonism can increase the effects of anti-angiogenic compounds. U87MG, U251, and T98G bearing nude mice were treated with bevacizumab (4 mg/kg iv every 4 days) and sunitinib (40 mg/kg po qd) alone or in combination with or PRX177561 (50 mg/kg po qd) in subcutaneous xenograft models. Tumors were randomized when they had reached a volume of 100–150 mm^3^ and then treated for 35 days. Tumor sizes were measured every 2 days. After 35 days, animals were sacrificed and tumors were harvested and weighed. Time to progression (TTP) was calculated as described in [26]. The hazard ratios (HRs) were used as a parameter to compare treatments [39]. In Figs. 3 and 4, we show the effects observed in U251, U87MG, and T98G tumors when compared to bevacizumab (Fig. 3) or sunitinib (Fig. 4). PRX177561 and bevacizumab were able alone or in combination to reduce U87MG tumor weight and increase TTP values (Fig. 3a). The HR values for the rate of progression were significantly greater for the combination of bevacizumab with PRX177561 (9.98 compared to control) than for either agent alone (HRs of 2.70 and 1.96 for PRX177561 and bevacizumab, respectively, Fig. 3c). A similar pattern was seen with U251MG and T98G xenografts with reduced tumor weights and increased TTP values (Fig. 3). Once again, the tumor progression HRs for the combinations of PRX177561 with bevacizumab (HR 10.47 in U251MG tumors and 7.02 in T98G tumors) versus either agent alone was significantly greater (Fig. 3). It was also noticeable that the tumor weights after 35 days treatment with the combination showed very little growth from the initial 100–150 mm^3^, with a 80–90% reduction in tumor vessel density and Ki67 proliferation index and a sizeable increase in apoptosis (Fig. 5).

PRX177561 and sunitinib, alone or in combination, reduced the tumor weights of all three cell types by approximately 80%, reduced the tumor blood vessel density, and increased the TTP (Fig. 4). This was associated with the modulation of Ki67 proliferation index (Fig. 5a, b), increased apoptosis (Fig. 5c, d), and increased palisading necrosis (Fig. 5e, f). The combination of PRX177561 with sunitinib had significantly greater progression HR values than with either agent alone in all three cell lines (Fig. 4). The combination HR values compared to control were between 6.73 and 8.35.

### In vivo effects of CXCR4 antagonist, PRX177561, alone or in combination with bevacizumab or sunitinib in orthotopic intracranial models: DFS and OS parameters

Luciferase-expressing U87 cells were injected in female nude mice as described and 5 days after cell injection, and when no bioluminescent lesions were visible, animals were randomly assigned to one of six different treatment groups: (1) vehicle (control), (2) PRX177561, (3) bevacizumab, (4) PRX177561 plus bevacizumab, (5) sunitinib, and (6) sunitnib plus PRX177561. Since the brain to plasma ratio of PRX177561 measured at the Cmax value is close to 1.0, indicating a good permeability through the blood-brain barrier, we treated animals bearing orthotopic intra-brain tumors with the same PRX177561 dose used for subcutaneous xenografts. The disease-free survival period (DFS) was defined as the period when no bioluminescent signal was detectable. After 35 days, treatments were stopped and animals were followed for overall survival (OS) determination. Animals were sacrificed when signs of distress were noticed.

**Fig. 6:**
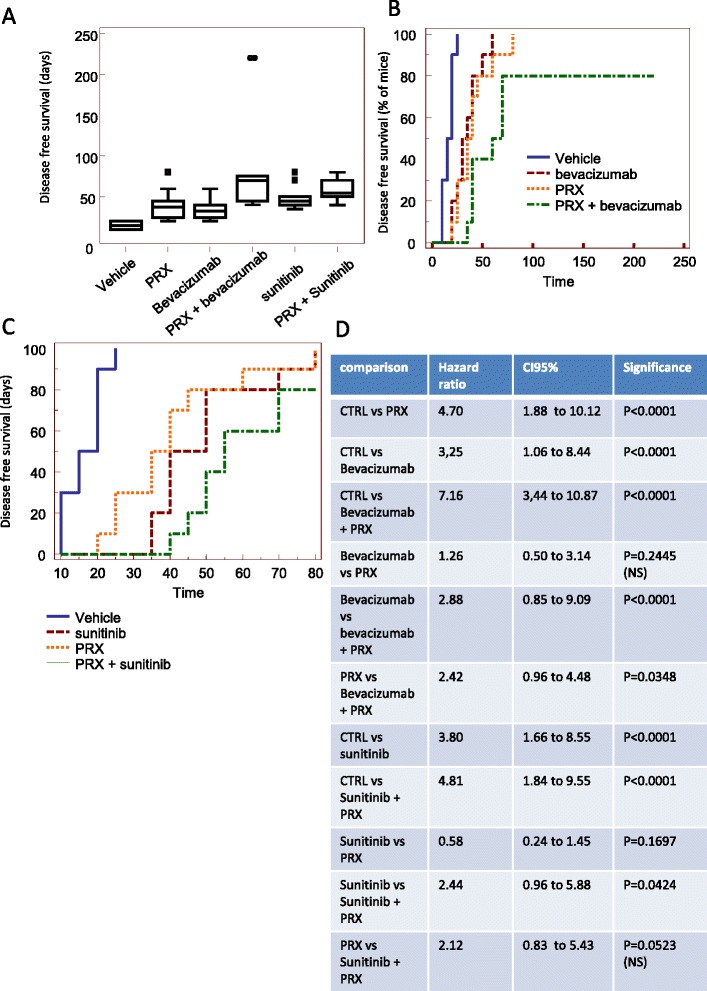
In vivo effects of CXCR4 antagonist, PRX177561, alone or in combination with bevacizumab or sunitinib in orthotopic intra-brain models: Disease-free survival (DFS) analyses. Luciferase-expressing U87 cells were injected in female nude mice as described, and 5 days after cell injection, when no bioluminescent lesions were visible, animals were randomly assigned to one of the six different treatment groups: (1) vehicle (control), (2) PRX177561, (3) bevacizumab, (4) PRX177561 plus bevacizumab, (5) sunitinib, and (6) sunitnib plus PRX177561. The presence of a bioluminescent signal was considered to define the DFS. After 35 days, treatments were stopped and animals were followed for the presence of bioluminescent positive lesions in order to calculate the DFS. **a** DSF graphical representation in our arms. **b** Kaplan-Meier rates analyzed for PRX177561 ± bevacizumab. **c** Kaplan-Meier rates analyzed for PRX177561 ± sunitinib. **d** Statistical analysis

Treatment with bevacizumab, sunitinib, and PRX177561 all increased the DFS period (by 2.2-, 3.1-, and 2.8-fold, respectively, Fig. 6a). The HR values are shown in Fig. 6d. In the combination dosing settings, PRX177561 further increased the DFS period with bevacizumab (by 2.1-fold) and sunitinib by 1.2-fold. Thus, in the bevacizumab plus PRX177561 combination, the DFS was 4.7-fold greater than that seen with the untreated control, and in the sunitinib plus PRX177561 3.7-fold.

The Kaplan-Meier curves (Fig. 6b, c) demonstrate the benefit of combining PRX177561 with bevacizumab and sunitinib.

**Fig. 7:**
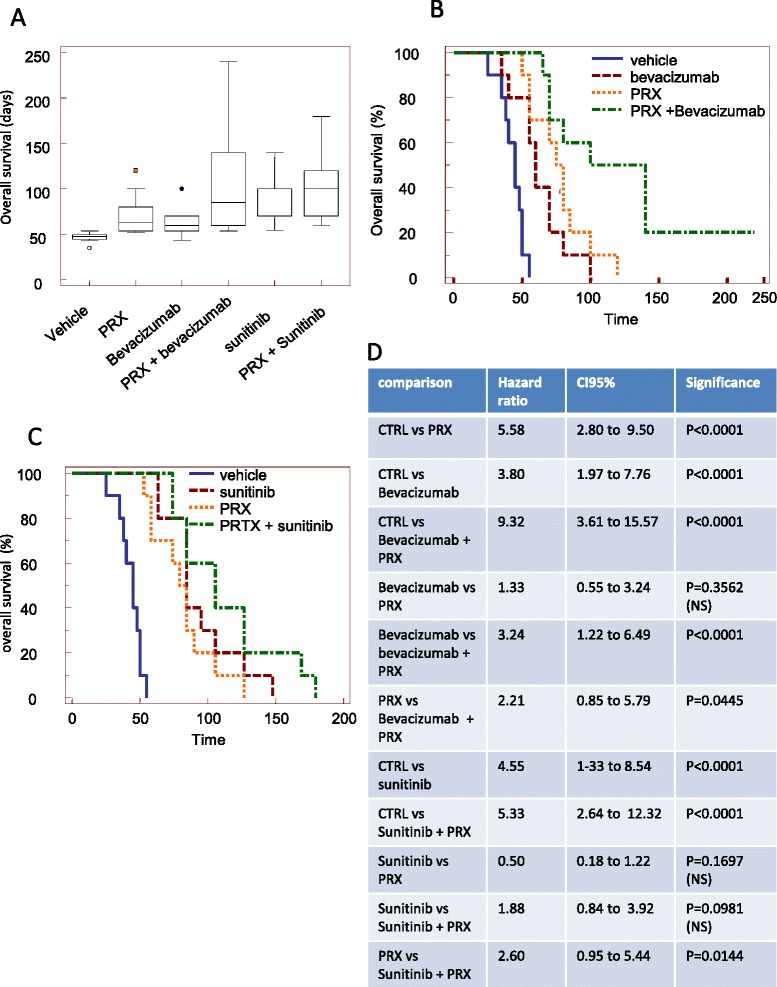
In vivo effects of CXCR4 antagonist, PRX177561, alone or in combination with bevacizumab or sunitinib in orthotopic intra-brain models: overall survival determinations. Luciferase-expressing U87 cells were injected in female nude mice as described, and 5 days after cell injection, when no bioluminescent lesions were visible, animals were randomly assigned to one of six different treatment groups: (1) vehicle (control), (2) PRX177561, (3) bevacizumab, (4) PRX177561 plus bevacizumab, (5) sunitinib, and (6) sunitnib plus PRX177561. The presence of a bioluminescent signal was considered to define the disease-free survival (DFS). After 35 days, treatments were stopped and animals were followed for overall survival (OS) determination. Animals were sacrificed when a sign of distress was noticed. **a** DFS graphical representation in our arms. **b** Kaplan-Meier rates analyzed for PRX177561 ± bevacizumab. **c** Kaplan-Meier rates analyzed for PRX177561 ± sunitinib. **d** Statistical analysis

Consistent with the inhibition of tumor growth, there was a significant increase in median overall survival (Fig. 7a) in PRX177561 treated animals from 47 to 71 days (1.5 times) when compared to control (*P* < 0.005) with a HR = 5.58. Bevacizumab increased OS by approximately 45% (1.45 times) with a HR = 3.80 when compared to untreated animals. Similarly, sunitinib increased OS by approximately 77% with a HR = 4.55 when compared to untreated animals. The addition of PRX-177561 to bevacizumab or sunitinib further increased median survival to 144 (2.3 times with HR = 3.24) and 107 (1.29 times, HR = 1.88) days, respectively. The analysis of Kaplan-Meier curves (Fig. 7b, c) indicated there was a benefit to the combination in the increase overall survival of PRX177561 both with bevacizumab (Fig. 7b) and sunitinib (Fig. 7c).

## Discussion

Despite extensive preclinical and clinical research, glioblastoma remains among the most devastating malignancies. Because glioblastomas are highly vascular tumors, therapies that target angiogenesis have generated substantial interest [3, 4]. The novel CXCR4 antagonist showing good brain penetration was demonstrated to reduce growth in vitro and in vivo of GBM cells and induce glioma stem cell differentiation [40]. So, we investigated the effect of PRX177561, with and without bevacizumab or sunitinib, by using subcutaneous and intracranial GBM cell inoculation in nude mice. Here, we observed that PRX177561 alone inhibited tumor growth and increased the efficacy of both bevacizumab and sunitinib resulting in a significant reduction in tumor growth in animal models of GBM. This is in agreement with previous experiments using AMD3100 [22, 38, 41–46] or POL5551 [21]. We show also that bevacizumab-mediated inhibition of tumor growth can be amplified by the simultaneous blockade of the CXCR4 and VEGFR. Due to the species selectivity of bevacizumab (i.e., only the influence of the tumor-derived VEGF would have been inhibited in these experiments, and the mouse stromal cell-derived VEGF would have been unaffected), we also investigated the effect of the small molecule inhibitor of the VEGFR2 receptor sunitinib in an attempt to block all VEGFR signaling. Somewhat surprisingly, there was no significant difference in the ability of bevacizumab and sunitinib to inhibit the growth of these tumors, suggesting that at least in these models, stromal cells play little part in promoting VEGF-mediated neo-angiogenesis of the growing tumors. However, since CXCR4 blockade also amplified the effect of sunitinib on the tumor growth, this provides extra evidence that the SDF-1α/CXCR4 axis plays a major role in promoting tumor growth. PRX177561 also enhanced survival in combination with both bevacizumab and sunitinib in the orthotopic model. Consistent with the report of Barone et al. [21], we observed that the combination a CXCR4 antagonist with an anti-VEGF antibody was able to reduce tumor growth and increase overall survival in intra-brain U87 xenografts. In this study, we showed that the combination of PRX177561 with bevacizumab, or sunitinib, increased median TTP values in subcutaneous xenografts and DFS and OS in intra-brain models when compared to single treatments. PRX177561 reduced the expression of Nestin in vivo indicating that CXCR4 antagonism also reduced the maintenance of the cancer stem cell population as previously demonstrated [40] It was interesting to note that the subcutaneous tumors less susceptible to bevacizumab and sunitinib (i.e., those larger tumors after treatment) expressed higher levels of SDF1α, CXCR4, and angiopoietin 2, after blockade of VEGFR signaling with these two agents. This is additional evidence these molecules may be associated with tumor resistance to anti-angiogenic therapy. The expression of SDF1α and CXCR4 has been strongly implicated in tumor growth, promoting cell migration and the recruitment of cells implicated in the revascularization process in tumors [17, 44–47]; this strongly suggested that SDF1/CXCR4 assists tumors in evading anti-angiogenic therapy. The microenvironment contribution in GBM development is increasingly emphasized. An interplay exists between CSCs, differentiated GBM cells, and the microenvironment, mainly through secreted chemokines causing recruitment of fibroblasts, endothelial, mesenchymal, and inflammatory cells to the tumor, via CXCR4. A favorable tumor microenvironment is also able to increase the growth of GBM-inducing cancer stem cells to enter in the cell cycle. Cancer stem cells (CSCs) or tumor-initiating cells (TICs) drive GBM development, invasiveness, and drug resistance [48]. It has been demonstrated that CSCs, isolated from human GBMs, express elevated levels of CXCR4 and release CXCL12 [49]. Possessing direct antitumor effects, mainly against cancer stem cell population, PRX177561 associates two fundamental properties to bevacizumab or sunitinib treatment.

### Limits of the study

A limit of this study could be that most of the work is done in a subcutaneous xenograft model, and only a small part done using an intracranial xenograft model. However, we have to consider that the subcutaneous xenograft model, the simplest existing murine model to study in vivo effects of therapeutic compounds in GBMs, shows good reproducibility of tumor genesis and synchronicity of experiments as widely considered in literature [28]. Subcutaneous tumors typically grow in a compact form whereas orthotopic tumors display an infiltrative growth. Therefore, the information obtained from both models is surely different. Subcutaneous models offer the opportunity to study in a simple manner mainly antitumor effects maintaining the opportune pharmacological selective pressure for the whole duration of the experiment, i.e., until the time of tumor sampling. Therefore, subcutaneous model is necessary for the histochemical, immunohistochemical, and molecular analyses with the possibility to address the question whether any phenotypic differences are surely due to treatments. We used the orthotopic model, instead, to verify whether the growth of a small amount of cells injected in the brain, mimicking the tumor recurrence after previous treatments (i.e., surgery), was influenced by examined pharmacological treatments. From this model, we considered two parameters: disease-free survival (DFS, as the time in which a bioluminescent positive event was measured for each tumor) and the overall survival (OS, as time elapsing from the inoculation of the cancer to euthanasia of animals after a single treatment cycle which was 35 days). We monitored animals for longer times (>200 days) when the pharmacological selective pressure was lost. It is a necessary state that we did not want to deal the animals for longer times since chronic treatments could have important side effects as suggest by clinical data by using different anti-target therapies (i.e., anti-her2 therapies).

## Conclusions

Our study, however, provides evidence for an enhanced survival effect on GBM-bearing mice which were treated with combination between PRX177561 and bevacizumab or sunitinib and represents a significant scientific rationale for clinical evaluation of this combined therapy that targets both VEGF/VEGFRs and CXCL12/CXCR4. PRX177561 is currently being assessed in a phase 1 clinical trial (NCT02765165).
